# Association between Serum 25-Hydroxyvitamin D Level and Stroke Risk: An Analysis Based on the National Health and Nutrition Examination Survey

**DOI:** 10.1155/2021/5457881

**Published:** 2021-10-29

**Authors:** Lan Wang, Shu Li, G. H. Anuja Sanika, Jinsheng Zhao, Hui Zhang, Lin Zhao, Wenfeng Wang

**Affiliations:** ^1^School of Nursing, Tianjin Medical University, Tianjin 300070, China; ^2^School of Public Health, Tianjin Medical University, Tianjin 300070, China; ^3^International Medical School, Tianjin Medical University, Tianjin 300070, China; ^4^Department of Acupuncture, Tianjin Nankai Hospital, Tianjin 300100, China; ^5^Department of Nephrology, Tianjin Hospital, Tianjin 300210, China; ^6^School of Science, Shanghai Institute of Technology, Shanghai 201418, China

## Abstract

**Background:**

To analyze the association between serum 25-hydroxyvitamin D level (25(OH)D) and stroke risk based on the National Health and Nutrition Examination Survey (NHANES).

**Methods:**

Between 2007 and 2018, the baseline information of participants from NHNES was collected. Univariate analysis was used to identify the covariates. Multivariate logistic regression model was used to analyze the association between serum 25(OH)D level and the stroke risk.

**Results:**

Of the 8,523 participants, there were 310 participants with stroke and 8,213 participants without stroke. The multivariate logistic analysis showed that serum 25(OH)D deficiency (odds ratio (OR): 1.993, 95% confidence intervals (CI): 1.141-3.481, and *P* = 0.012) was the significant risk factors for stroke. Subgroup analysis showed that non-Hispanic whites with serum 25(OH)D deficiency (OR: 2.501, 95% CI: 1.094-5.720, and *P* = 0.001) and insufficiency (OR: 1.853, 95% CI: 1.170-2.934, and *P* = 0.006) were associated with a higher risk of stroke than those with normal 25(OH)D levels.

**Conclusions:**

Serum 25(OH)D deficiency may be associated with an increased risk of stroke.

## 1. Introduction

Stroke remains the third most common cause of disability and the second most common cause of death worldwide [1]. In the United States, there were an estimated 795,000 new or recurrent stroke with approximately 130,000 deaths due to stroke each year [2]. The prevalence increases with age in both females and males. By 2030, an additional 3,400,000 adults are estimated to have a stroke in the United States, which increased by 20.5% with 2012 [3]. Stroke is associated with modifiable risk factors (hypertension, hyperglycemia, obesity, hyperlipidemia, and renal dysfunction) and behavioral risk factors (sedentary lifestyle, cigarette smoking, and unhealthy diet) [2, 4]. Interestingly, vitamin D (25-hydroxyvitamin D, 25(OH)D), a hormone mainly regulating calcium homeostasis, is found to be associated with the development of various nonskeletal chronic diseases, including stroke [5], cardiovascular disease [6], cancer [7], metabolic disorder [8], autoimmune disease [9], and infectious diseases [10].

In recent years, several studies have been conducted on the association between 25(OH)D level and stroke risk, but the results are inconsistent. Zhou et al. reported that 25(OH)D levels were associated with ischemic stroke (relative risk: 2.45) [11]. Berghout et al. also found a correlation between 25(OH)D level and prevalent stroke (adjusted odds ratio (OR): 1.31), but only extremely low 25(OH)D level was related to incident stroke (hazard ratio (HR): 1.25), showing that low 25(OH)D level may not increase the risk of stroke [12]. There is another evidence suggesting no association between 25(OH)D levels and incidence of stroke (HR: 1.00) [13]. In view of the inconsistent results, in this study, we used a pooled cross-sectional data from the National Health and Nutrition Examination Survey (NHANES) (2007-2018) to further analyze the association of serum 25(OH)D level with stroke risk.

## 2. Methods

### 2.1. Data Sources

NHANES, a nationally representative survey for noninstitutionalized civilians in the United States, is conducted in two-year cycles, with approximately 10,000 persons in each cycle [14]. Participants aged over 18 years who had serum 25(OH)D level measured during the survey were enrolled in this study. Pregnant women and participants who did not respond to the question on stroke history and had missing information (e.g., age, sex, marital status, family income, educational level, and condition of complications) were excluded from the study.

The data used in this study were accessed from NHANES, a continuous program performed by the National Center for Health Statistics. The approval from the Institutional Review Board of Tianjin Medical University was not required because the data from NHANES were freely available.

The baseline characteristics of participants were collected, including age, gender, body mass index (BMI), race (Mexican Americans, non-Hispanic whites, non-Hispanic blacks, other races), marital status (married, widowed/divorced, unmarried, and living with partner), family income (<20,000$ and ≥20,000$), educational level (<high school, high school, and >high school), dietary intake (dietary fiber, total fat, fruits, vegetables, and vitamins A, B, C, and E), total cholesterol (TC), glycated hemoglobin (GHb), high-density lipoprotein (HDL), C-reactive protein (CRP), and 25(OH)D levels (deficiency: <30 nmol/L, insufficiency: 30-50 nmol/L, normal: 50-125 nmol/L, and adequacy: >125 nmol/L), as well as presence or absence of drinking, emphysema, chronic bronchitis, hypertension, high cholesterol, and diabetes mellitus.

The stroke was determined based on the Medical Condition Questionnaire (MCQ). Question MCQ160f “Has a doctor or other health professional ever told you that you had a stroke?” was asked by interviews. The participants answered “yes” were deemed to have stroke.

Diabetes mellitus was identified through the Diabetes Questionnaire (DIQ). Question DIQ010 is “Other than during pregnancy, have you ever been told by a doctor or health professional that you have diabetes or sugar diabetes?” Participants who answered “yes” were considered as diabetic.

The Blood Pressure & Cholesterol Questionnaire (BPQ) question BPQ080 is “Have you ever been told by a doctor or other health professional that your blood cholesterol level was high?” Participants who answered “yes” were considered as with high cholesterol level.

Hypertension was determined according to the question BPQ020, “Have you ever been told by a doctor or other health professional that you had hypertension, also called high blood pressure?” Participants who answered “yes” were considered with hypertension.

Dietary intake was estimated by two 24-hour dietary recall, a validated Automated Multiple-Pass Method jointly completed by the United States Department of Agriculture (USDA) and the United States Department of Health and Human Services (DHHS) [15]. The specific intake of each nutrient was available in the Dietary Interview-Total Nutrients Intakes. Consumptions of dietary fiber, total fat, fruits, vegetables, vitamin A, vitamin B, vitamin C, and vitamin E were retrieved from the dietary data.

### 2.2. Measurement of Serum 25(OH)D Level

Serum 25(OH)D level (ng/mL) was thought to be the optimal indicator to assess vitamin D status [16]. In the NHANES (2001-2006), the serum 25(OH)D level in approximately 88% of the adult participants was measured using radioimmunoassay kits (DiaSorin Inc., Stillwater, MN). An independent calibration was conducted for radioimmunoassay kits against high-performance liquid chromatography-purified 25(OH)D. Since 2007, the serum 25(OH)D level was measured using ultrahigh performance liquid chromatography-tandem mass spectrometry. Due to the differences in the results of these two measurement methods, this study only analyzed data from 2007 to 2018. The detailed measurement methods and quality assurance for serum 25(OH)D level could be found in the survey laboratory data [17]. The Institute of Medicine (IOM) and United State Preventive Services Task Force define vitamin D sufficiency as a total 25(OH)D level greater than 50 nmol/L [18, 19]. In this study, serum 25(OH)D level < 30 nmol/L is thought as deficiency, 30-50 nmol/L as insufficiency, 50-125 nmol/L as the normal value, and >125 nmol/L as adequacy.

### 2.3. Statistical Analysis

The SAS software (version 9.4, SAS Institute Inc., NC, USA) was employed to analyze the data. Normally, distributed data were represented as mean ± standard error (SE) and compared using *t* test, while nonnormal data was presented as median and quartile (M (Q1 and Q3)) and compared using Mann-Whitney *U* rank-sum test. *χ*^2^  test or Fisher's exact test was used to compare the enumeration data which were described as *n* (%). In the NHANES 2007–2018 study, exam weight was taken into account.

All included data from 2007 to 2018 were not missing. The covariates with significant difference in the univariate analysis were enrolled into the multivariate logistic regression model to analyze the association between serum 25(OH)D levels and the stroke risk. The difference was significant at *P* < 0.05.

## 3. Results

### 3.1. Basic Characteristics of Participants

**Figure 1:**
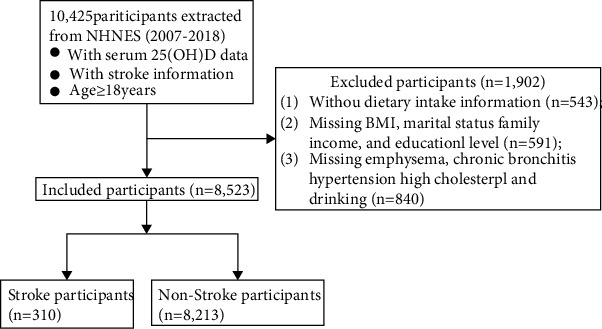
Flow chart of study population inclusion.

A total of 10,425 participants with serum 25(OH)D data, stroke information, and age ≥ 18 years were retrieved from the NHANES between 2007 and 2018. After excluding 543 participants without dietary intake data and 1,359 participants with other missing information (BMI, marital status, drinking, emphysema, chronic bronchitis, etc.), 8,523 participants were finally eligible for the study. The process of inclusion and exclusion of participants is shown in Figure 1. Of these 8,523 participants, the mean age was 46.96 ± 0.35 years, 4,201 (48.60%) were males, and 4,322 (51.40%) were females. Among included participants, 1,519 (8.31%) were Mexican Americans, 4,294 (71.57%) were non-Hispanic whites, 1,511 (10.25%) were non-Hispanic blacks, and 1,199 (9.87%) were other races. In addition, there were 749 (6.24%) participants of serum 25(OH)D deficiency, 2,026 (18.71%) participants of serum 25(OH)D insufficiency, 5,585 (72.40%) participants of serum 25(OH)D normal, and 163 (2.65%) participants of serum 25(OH)D adequacy. There were 310 participants of stroke and 8,213 participants of no stroke. More detailed characteristics of participants are shown in Table 1.

### 3.2. Comparison of the Baseline Characteristics between the Stroke and No Stroke Groups

**Figure 2:**
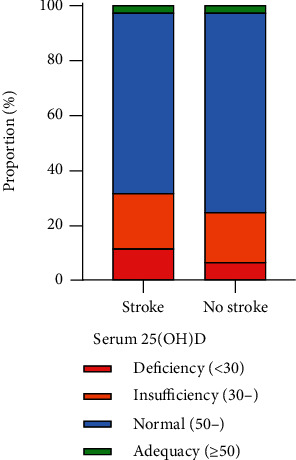
The serum 25(OH)D level distribution of stroke and no stroke groups.

Univariable analyses are shown in Table 2. The results indicated that age (*t* = −13.62, *P* < 0.001), BMI (*t* = −3.60, *P* < 0.001), GHb (*t* = −6.67, *P* < 0.001), CRP (*t* = −3.55, *P* < 0.001), and the proportion of drinking (*χ*^2^ = 25.510, *P* < 0.001), emphysema (*χ*^2^ = 42.304, *P* < 0.001), chronic bronchitis (*χ*^2^ = 61.132, *P* < 0.001), hypertension (*χ*^2^ = 194.882, *P* < 0.001), high cholesterol (*χ*^2^ = 28.263, *P* < 0.001), and diabetes mellitus (*χ*^2^ = 168.476, *P* < 0.001) of participants in the stroke group were higher than those in the no stroke group. However, participant's education level (*χ*^2^ = 37.712, *P* < 0.001), HDL (*t* = 2.97, *P* = 0.006), serum 25(OH)D (*χ*^2^ = 9.357, *P* = 0.025), the proportion of family income (*χ*^2^ = 14.866, *P* < 0.001), intake of dietary fiber (*t* = 3.36, *P* = 0.002), total fat (*t* = 3.04, *P* = 0.005), vegetables (*t* = 3.14, *P* = 0.004), vitamin B (*t* = 4.06, *P* < 0.004), and vitamin E (*t* = 3.28, *P* = 0.003) were lower in the stroke group compared with the no stroke group. In addition, there were statistical differences in the race (*χ*^2^ = 12.177, *P* = 0.007) and marital status (*χ*^2^ = 25.660, *P* < 0.001) between the two groups. The serum 25(OH)D level distribution of stroke and no stroke groups is shown in Figure 2, and the results indicated that the proportion of patients with serum 25(OH)D deficiency and insufficiency in the stroke group was higher in the stroke group than that in the no stroke group.

### 3.3. Relationship between Serum 25(OH)D Level and Stroke Risk

**Figure 3:**
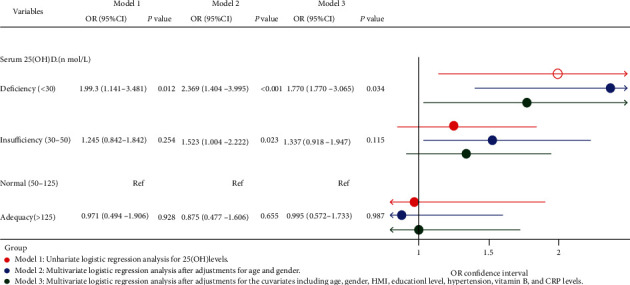
Multivariate logistic regression forest plot of the association between serum 25(OH)D levels and the stroke risk.

Multivariate logistic regression analysis results of the association between serum 25(OH)D levels and the stroke risk are summarized in Figure 3. As is shown, in the univariate serum 25(OH)D logistic regression analysis (model 1), the patients with serum 25(OH)D deficiency (OR: 1.993, 95% confidence interval (95% CI): 1.141-3.481, and *P* = 0.012) had an increased risk of stroke in contrast to those with normal 25(OH)D levels. After adjustment for covariates of age and gender (model 2), the risk of stroke was increased by 1.369- and 0.523-fold, respectively, in patients with serum 25(OH)D deficiency (OR: 2.369, 95% CI: 1.404-3.995, and *P* < 0.023) and insufficiency (OR: 1.523, 95% CI: 1.044-2.222, and *P* = 0.023) when compared with those with normal serum 25(OH)D levels. After the adjustment of all the covariates (model 3), such as age, gender, BMI, educational level, hypertension, vitamin E, and CRP levels, serum 25(OH)D deficiency (OR: 1.770, 95% CI: 1.023-3.065, and *P* = 0.034) was still the independent risk factors for stroke.

### 3.4. Further Analysis of the Relationship between Serum 25(OH)D Level and Stroke Risk Based on Race

Further analysis results of the relationship between serum 25(OH)D levels and stroke risk based on race are shown in Table 3. Except for non-Hispanic whites, the association between serum 25(OH)D level and stroke risk was not statistically significant among Mexican Americans, non-Hispanic blacks, and other races (all *P* > 0.05). In the univariate serum 25(OH)D logistic regression analysis, the non-Hispanic whites with serum 25(OH)D deficiency (OR: 4.651, 95% CI: 1.908-11.338, and *P* < 0.001) and insufficiency (OR: 1.957, 95% CI: 1.257-3.047, and *P* = 0.002) had a higher risk of stroke than those with normal 25(OH)D levels. After adjustment for part covariates, the risk of stroke was increased by 2.087- and 0.982-fold, respectively, in patients with serum 25(OH)D deficiency (OR: 3.087, 95% CI: 1.390-6.856, and *P* = 0.004) and insufficiency (OR: 1.982, 95% CI: 95% CI: 1.246-3.153, and *P* = 0.003) as compared with participants with normal serum 25(OH)D levels. After adjustment for all the covariates, the risk of stroke in patients with serum 25(OH)D deficiency (OR: 2.501, 95% CI: 1.094-5.720, and *P* = 0.001) and insufficiency (OR: 1.853, 95% CI: 1.170-2.934, and *P* = 0.006) was 2.501 times and 1.853 times that of patients with normal serum 25(OH)D levels, respectively.

## 4. Discussion

A total of 8,523 eligible participants were involved into the present study, among whom 310 participants were subjected to stroke, while 8,213 participants were not. The multivariate logistic analysis showed that serum 25(OH)D deficiency was the significant risk factor for stroke. All these findings suggested that the patients with serum 25(OH)D deficiency (<30 nmol/L) might have an increased risk of stroke. Subgroup analysis showed that non-Hispanic whites with serum 25(OH)D deficiency and insufficiency were associated with a high risk of stroke.

As a fat-soluble vitamin, vitamin D, can affect cardiomyocytes, endothelial cells, vascular smooth muscle cells, and inflammatory cells by binding vitamin D receptors (VDR) and exert the effects of inhibiting myocardial hypertrophy, protecting vascular endothelium, and regulating inflammatory responses, consequently decreasing the onset risk of cardiovascular and cerebrovascular diseases and improving patients' prognosis [20–22]. 25(OH)D, a major circulation form of vitamin D in the body, can better reflect vitamin D status. Several studies reported vitamin D deficiency, whether in serum or in intake, may be associated with an increased risk of ischemic stroke [11, 23]. In the present study, a pooled cross-sectional data from NHANES were used to identify the association of serum 25(OH)D level with the stroke risk. The risk of stroke was found to significantly increase in patients with serum 25(OH)D deficiency and insufficiency. However, vitamin D status is under the influence of various factors including age, living areas, exposure to sunlight, and vitamin D daily dietary intake [24, 25]. After adjustment for multiple covariates, our results still showed that serum 25(OH)D deficiency is related to an increased risk of stroke.

The impact of serum 25(OH)D level-related stroke risk between different races is controversial. The study of Judd et al. indicated that lower 25(OH)D level is a significant risk factor for incident stroke, and no statistically significant difference was observed between blacks and whites [26]. However, Michos et al. found that serum 25(OH)D deficiency was associated with a higher risk of stroke in whites but not in blacks (hazard ratios 2.13 vs. 0.93) [27]. Our results showed that a lower serum 25(OH)D level was related to an increased risk of stroke in non-Hispanic whites. Robinson-Cohen et al. also demonstrated that lower serum 25(OH)D level was related to an increased risk of incident coronary heart disease among participants who were Hispanic [28]. In addition, several studies indicated that both low and high serum 25(OH)D levels have increased the risk of stroke [29, 30]. However, the relationship between high 25(OH)D levels and stroke risk was not observed in this study. The possible explanation was that the sample size of participants with higher high 25(OH)D levels is not large, and no statistically significant results can be obtained.

At present, the effect of 25(OH)D level on the stroke risk can be explained by several mechanisms below. Inappropriate activation of the renin-angiotensin system (RAS) may be a major risk factor for stroke [31]. 25(OH)D, a negative endocrine regulator of the renin-angiotensin system (RAS), may influence the stroke risk through RAS regulation [31, 32]. A previous experiment showed that by regulating cholesterol efflux and macrophage polarization through elevated CYP27A1 activation, vitamin D played a protective role against atherosclerosis in hypercholesterolemic swine [33]. Activated vitamin D may defer atherosclerosis by inhibiting the formation of foam cells and the process of macrophage cholesterol absorption, consequently reducing the risk of developing stroke [34]. There is another study showing that vitamin D deficiency contributes to facilitating secondary hyperparathyroidism, while the increased levels of parathyroid hormone may accelerate the inflammatory response in atherosclerosis [35, 36]. In addition, activated vitamin D plays a crucial role in preventing thrombosis, which may explain why the low vitamin D level is associated with an increased risk of ischemic stroke [37, 38].

The strength of the present study was that it was a large-scale, population-based study, providing strong evidence for assessing the relationship between vitamin D status and stroke risk. Compared with the single-center studies, our research results may be more generalizable. Furthermore, we conducted a further analysis of the relationship between serum 25(OH)D level and stroke risk based on race. However, there existed some limitations. First, the data in our study were accessed from the NHNES database, which may be lack of some important variables, such as vitamin D supplementation, exposure to sunlight, living areas, and seasons of vitamin D measurement. Second, the stroke history was confirmed through self-reported data. Despite lack of verification for self-reporting stroke in NHANES, the stroke history from questionnaire was checked. In several studies, the self-reported data from NHANES were used to identify the risk factors for cardiovascular diseases [39–41].

## 5. Conclusions

The results suggested that serum 25(OH)D deficiency (<30 nmol/L) might be related to an increased risk of stroke. In addition, non-Hispanic whites with serum 25(OH)D deficiency (<30 nmol/L) and insufficiency (30-50 nmol/L) were associated with a high risk of stroke than those with normal 25(OH)D levels.

## Figures and Tables

**Table 1:** Baseline characteristics of participants after multiple imputation.

Variables	Description (*n* = 8,523)
Age, years, mean ± SE	46.96 ± 0.35
Gender, *n* (%)	
Male	4,201 (48.60)
Female	4,322 (51.40)
BMI, kg/m^2^, mean ± SE	28.79 ± 0.10
Race, *n* (%)	
Mexican Americans	1,519 (8.31)
Other races	1,199 (9.87)
Non-Hispanic whites	4,294 (71.57)
Non-Hispanic blacks	1,511 (10.25)
Marital status, *n* (%)	
Married	4562 (57.47)
Widowed/divorced	1913 (17.89)
Unmarried	1377 (16.93)
Living with partner	671 (7.71)
Educational level, *n* (%)	
<high school	2408 (18.46)
High school	2010 (23.56)
>high school	4105 (57.98)
Family income, *n* (%)	
<20,000$	2100 (16.96)
≥20,000$	6423 (83.04)
Drinking, *n* (%)	2337 (23.04)
Emphysema, *n* (%)	203 (1.91)
Chronic bronchitis, *n* (%)	474 (5.15)
Hypertension, *n* (%)	3022 (30.53)
High cholesterol, *n* (%)	3547 (39.33)
Diabetes mellitus, *n* (%)	1045 (8.63)
Dietary intake, mean ± SE	
Dietary fiber	16.68 ± 0.28
Total fat	82.96 ± 0.81
Fruits	1.00 ± 0.02
Vegetables	1.58 ± 0.03
Vitamin A	632.18 ± 10.22
Vitamin B	2.23 ± 0.02
Vitamin C	84.52 ± 1.96
Vitamin E	7.90 ± 0.12
TC, nmol/L, mean ± SE	196.89 ± 0.71
GHb, nmol/L, mean ± SE	5.60 ± 0.02
HDL, nmol/L, mean ± SE	52.72 ± 0.37
CRP, nmol/L, mean ± SE	0.38 ± 0.01
25(OH)D, nmol/L, *n* (%)	
Deficiency (<30)	749 (6.24)
Insufficiency (30-50)	2,026 (18.71)
Normal (50-125)	5,585 (72.40)
Adequacy (≥125)	163 (2.65)
Stroke, *n* (%)	
Yes	8,213 (97.20)
No	310 (2.80)

Notes: BMI: body mass index; TC: total cholesterol; GHb: glycated hemoglobin; HDL: high-density lipoprotein; CRP: C-reactive protein; 25(OH)D: 25-hydroxyvitamin D. Values were presented as mean ± standard error (SE) or median and quartile (M (Q1 and Q3)) for continuous variables and *n* (%) for categorical variables. In the NHANES 2007–2018 study, exam weight was taken into account.

**Table 2:** Comparison of the baseline characteristics between stroke and nonstroke groups.

Variables	Nonstroke group (*n* = 8,213)	Stroke group (*n* = 310)	Statistic	*P*
Age, years, mean ± SE	46.47 ± 0.35	63.93 ± 1.28	*t* = −13.62	<0.001
Gender, *n* (%)			*χ* ^2^ = 1.246	0.264
Male	4045 (48.74)	156 (44.01)		
Female	4168 (51.26)	154 (55.99)		
BMI, kg/m^2^, mean ± SE	28.75 ± 0.10	30.16 ± 0.36	*t* = −3.60	0.001
Race, *n* (%)			*χ* ^2^ = 12.177	0.007
Mexican Americans	1492 (8.44)	27 (3.78)		
Other races	1172 (9.96)	27 (6.68)		
Non-Hispanic whites	4108 (71.44)	186 (76.23)		
Non-Hispanic blacks	1441 (10.16)	70 (13.31)		
Marital status, *n* (%)			*χ* ^2^ = 25.660	<0.001
Married	4396 (57.59)	166 (53.30)		
Widowed/divorced	1804 (17.48)	109 (32.20)		
Unmarried	1353 (17.20)	24 (7.71)		
Living with partner	660 (7.74)	11 (6.80)		
Educational level, *n* (%)			*χ* ^2^ = 37.712	<0.001
<high school	2291 (18.13)	117 (29.97)		
High school	1931 (23.40)	79 (29.04)		
>high school	3991 (58.47)	114 (40.99)		
Family income, *n* (%)			*χ* ^2^ = 14.866	<0.001
<20,000$	1989 (16.68)	111 (26.77)		
≥20,000$	6224 (83.32)	199 (73.23)		
Drinking, *n* (%)	2228 (22.66)	109 (36.32)	*χ* ^2^ = 25.510	<0.001
Emphysema, *n* (%)	179 (1.75)	24 (7.22)	*χ* ^2^ = 42.304	<0.001
Chronic bronchitis, *n* (%)	431 (4.79)	43 (17.71)	*χ* ^2^ = 61.132	<0.001
Hypertension, *n* (%)	2773 (29.23)	249 (75.83)	*χ* ^2^ = 194.882	<0.001
High cholesterol, *n* (%)	3364 (38.86)	183 (55.70)	*χ* ^2^ = 28.263	<0.001
Diabetes mellitus, *n* (%)	939 (8.00)	106 (30.64)	*χ* ^2^ = 168.476	<0.001
Dietary intake, mean ± SE				
Dietary fiber	16.74 ± 0.28	14.48 ± 0.71	*t* = 3.36	0.002
Total fat	83.32 ± 0.76	70.44 ± 4.55	*t* = 3.04	0.005
Fruits	1.00 ± 0.03	0.93 ± 0.08	*t* = 0.92	0.366
Vegetables	1.59 ± 0.03	1.38 ± 0.07	*t* = 3.14	0.004
Vitamin A	633.17 ± 10.04	597.84 ± 41.66	*t* = 0.89	0.381
Vitamin B	2.24 ± 0.02	1.89 ± 0.09	*t* = 4.06	<0.001
Vitamin C	84.80 ± 1.97	74.84 ± 5.03	*t* = 2.00	0.054
Vitamin E	7.93 ± 0.11	6.71 ± 0.41	*t* = 3.28	0.003
TC, nmol/L, mean ± SE	197.05 ± 0.72	191.52 ± 3.95	*t* = 1.37	0.179
GHb, nmol/L, mean ± SE	5.58 ± 0.01	6.09 ± 0.08	*t* = −6.67	<0.001
HDL, nmol/L, mean ± SE	52.81 ± 0.37	49.53 ± 1.05	*t* = 2.97	0.006
CRP, nmol/L, mean ± SE	0.37 ± 0.01	0.67 ± 0.09	*t* = −3.55	0.001
25(OH)D, nmol/L, *n* (%)			*χ* ^2^ = 9.357	0.025
Deficiency (<30)	715 (6.11)	34 (11.01)		
Insufficiency (30-50)	1957 (18.64)	69 (21.00)		
Normal (50-125)	5385 (72.59)	200 (65.66)		
Adequacy (≥125)	156 (2.66)	7 (2.34)		

Notes: BMI: body mass index; TC: total cholesterol; GHb: glycated hemoglobin; HDL: high-density lipoprotein; CRP: C-reactive protein; 25(OH)D: 25-hydroxyvitamin D. Values were presented as mean ± standard error (SE) or median and quartile (M (Q1 and Q3)) for continuous variables and *n* (%) for categorical variables. In the NHANES 2007–2018 study, exam weight was taken into account.

**Table 3:** Subgroup analysis of the association between serum 25(OH)D levels and the stroke risk based on race.

Race	*N* (%)	Model 1 OR (95% CI)	*P*	Model 2 OR (95% CI)	*P*	Model 3 OR (95% CI)	*P*
Mexican Americans							
25(OH)D, nmol/L							
Deficiency (<30)	115 (7.75)	0.762 (0.230-2.524)	0.643	1.015 (0.310-3.320)	0.979	0.868 (0.282-2.671)	0.798
Insufficiency (30-50)	524 (34.98)	0.676 (0.308-1.484)	0.309	0.786 (0.364-1.697)	0.522	0.778 (0.351-1.727)	0.520
Normal (50-125)	878 (57.17)	Ref		Ref		Ref	
Adequacy (≥125)	2 (0.10)	-		-		-	
Non-Hispanic whites							
25(OH)D, nmol/L							
Deficiency (<30)	108 (10.62)	4.651 (1.908-11.338)	<0.001	3.087 (1.390-6.856)	0.004	2.501 (1.094-5.720)	0.024
Insufficiency (30-50)	353 (29.13)	1.957 (1.257-3.047)	0.002	1.982 (1.246-3.153)	0.003	1.853 (1.170-2.934)	0.006
Normal (50-125)	731 (59.69)	Ref		Ref		Ref	
Adequacy (≥125)	7 (0.56)	0.954 (0.456-1.995)	0.897	0.922 (0.481-1.767)	0.800	1.011 (0.574-1.781)	0.969
Non-Hispanic blacks							
25(OH)D, nmol/L							
Deficiency (<30)	123 (2.40)	0.746 (0.437-1.273)	0.262	1.009 (0.550-1.851)	0.976	0.837 (0.470-1.492)	0.528
Insufficiency (30-50)	567 (12.43)	0.555 (0.209-1.473)	0.218	0.739 (0.276-1.977)	0.529	0.702 (0.264-1.864)	0.459
Normal (50-125)	3461 (81.65)	Ref		Ref		Ref	
Adequacy (≥125)	143 (3.53)	1.739 (0.185-16.329)	0.613	0.819 (0.097-6.899)	0.848	1.298 (0.145-11.605)	0.808
Other races							
25(OH)D, nmol/L							
Deficiency (<30)	403 (27.69)	0.615 (0.135-2.798)	0.513	1.050 (0.298-3.695)	0.937	0.828 (0.267-2.571)	0.734
Insufficiency (30-50)	582 (39.36)	0.334 (0.089-1.261)	0.092	0.408 (0.107-1.555)	0.172	0.379 (0.095-1.502)	0.151
Normal (50-125)	515 (32.36)	Ref		Ref		Ref	
Adequacy (≥125)	11 (0.60)	-		-		-	

Notes: model 1 is the univariate logistic regression analysis for 25(OH)D levels; model 2 is the multivariate logistic regression analysis after adjustments for age and gender; model 3 is the multivariate logistic regression analysis after adjustment for the covariates including age, gender, BMI, educational level, hypertension, vitamin B, and CRP levels.

## Data Availability

The data utilized to support the findings are available from the corresponding authors upon request.
